# CAR-Based Strategies beyond T Lymphocytes: Integrative Opportunities for Cancer Adoptive Immunotherapy

**DOI:** 10.3390/ijms20112839

**Published:** 2019-06-11

**Authors:** Ramona Rotolo, Valeria Leuci, Chiara Donini, Anna Cykowska, Loretta Gammaitoni, Giovanni Medico, Giorgio Valabrega, Massimo Aglietta, Dario Sangiolo

**Affiliations:** 1 Department of Oncology, University of Torino, 10140 Torino, Italy; ramona.rotolo@ircc.it (R.R.); valeria.leuci@ircc.it (V.L.); chiara.donini@ircc.it (C.D.); a.cykowska@gmail.it (A.C.); giovanni.medico@edu.unito.it (G.M.); giorgio.valabrega@ircc.it (G.V.); massimo.aglietta@ircc.it (M.A.); 2 Candiolo Cancer Institute FPO-IRCCS, 10060 Candiolo TO, Italy; loretta.gammaitoni@ircc.it

**Keywords:** CAR, adoptive immunotherapy, γδT, NK, NKT, CIK

## Abstract

Chimeric antigen receptor (CAR)-engineered T lymphocytes (CAR Ts) produced impressive clinical results against selected hematological malignancies, but the extension of CAR T cell therapy to the challenging field of solid tumors has not, so far, replicated similar clinical outcomes. Many efforts are currently dedicated to improve the efficacy and safety of CAR-based adoptive immunotherapies, including application against solid tumors. A promising approach is CAR engineering of immune effectors different from αβT lymphocytes. Herein we reviewed biological features, therapeutic potential, and safety of alternative effectors to conventional CAR T cells: γδT, natural killer (NK), NKT, or cytokine-induced killer (CIK) cells. The intrinsic CAR-independent antitumor activities, safety profile, and ex vivo expansibility of these alternative immune effectors may favorably contribute to the clinical development of CAR strategies. The proper biological features of innate immune response effectors may represent an added value in tumor settings with heterogeneous CAR target expression, limiting the risk of tumor clonal escape. All these properties bring out CAR engineering of alternative immune effectors as a promising integrative option to be explored in future clinical studies.

## 1. Introduction

T lymphocytes genetically redirected with antitumor chimeric antigen receptors (CARs) represent an innovative frontier of cancer adoptive immunotherapy. CARs are synthetic biology constructs generated by fusing the single-chain variable fragment (scFv) of a tumor-reactive monoclonal antibody with the T cell receptor (TCR) CD3 zeta chain, combined with additional costimulatory molecules [1,2,3,4]. CARs do not require antigen processing and human leucocytes antigen (HLA) presentation of their targets [5], which poses an important issue compared to the conventional TCRs in respect to the possible HLA downregulation by tumor cells. CAR-engineered T lymphocytes (CAR-T) against the B-lineage antigen CD19 recently produced impressive clinical results in the field of hematologic B-cell malignancies [6,7], including non-Hodgkin lymphoma, chronic lymphocytic leukemia (CLL) [8,9], and acute lymphoblastic leukemia (ALL) [10,11,12].

Clinical responses and improved survivals were paralleled with important concerns about possible toxicities mostly due to on-target off-tumor effects or cytokine release syndrome (CRS) [13,14,15].

These important clinical successes have, so far, not been replicated against advanced solid tumors, where crucial challenges are the identification of tumor-exclusive CAR targets and overcoming barriers by the immunosuppressive microenvironment [16,17,18].

Research efforts are currently dedicated to improve the safety and efficacy of CAR-based adoptive immunotherapies, including their application to patients with solid tumors.

Within this perspective, an intriguing approach is the alternative or integrative CAR engineering of immune effectors different from conventional αβ T lymphocytes.

Preclinical data, and even initial clinical studies, are exploiting different types of lymphocytes, like γδ T, Natural Killer (NK), NKT and Cytokine-induced Killer (CIK) cells as innovative platforms for CAR engineering. The biological features of such immune effectors, mainly their intrinsic CAR-independent antitumor activities, safety profile and ex vivo expansibility, may favorably contribute to the clinical development of CAR strategies including their extension to the field of solid tumors.

Here we review the main cell types currently explored as potential alternatives to conventional CAR-Ts. We discuss the main underlying rationale to explore different types of lymphocytes, their potential advantage and limitations, initial preclinical data, and a preview of pilot/ongoing clinical trials.

## 2. CAR γδT CELLS

### 2.1. Biological Features and Therapeutic Potential of γδT Cells

γδT lymphocytes are a highly conserved and distinct lineage of T cells and the first lineage generated during fetal development [19]. Unlike conventional T cells (T cells expressing αβTCR), γδT lymphocytes constitute approximately 1%–10% of the CD3+ T cells population in the peripheral blood cells and even a smaller fraction (1%–5%) in the lymphoid organs. They represent a major subset of resident T cells (10%–100%) in the epidermis of the skin, mucosa of the gastrointestinal tract, and the reproductive system [20]. γδT lymphocytes share many cell surface proteins and effector capabilities with cells of the innate immune system, such as NK cells. They are participating in the first line of defense that protects the host from microbial infections and cancer. Their preferential distribution in tissues favors their initial in situ defensive activity. γδT cells are considered unconventional since they typically do not express either of the CD4 or CD8 co-receptors. Unlike conventional αβ T cells, their TCRs are relatively invariant. γδT cells do not require antigen presentation by MHC complex. Their activation relies on cell-to-cell contact with APC. γδT lymphocytes can recognize stress inducible molecule such as MHC class I–related chain A and B (MICA/B) and non-peptide metabolites of the isoprenoid biosynthesis such as the cholesterol precursor isopentyl pyrophosphate, which is normally overproduced in cancer cells [21]. Artificial interference with the cholesterol synthesis pathway using the Food and Drug Administration (FDA)-approved drug zoledronic acid (ZA) on peripheral blood mononuclear cells (PBMCs) results in a surface accumulation of isopentyl pyrophosphate and subsequent stimulation of γδT cells. Overall, they present an abundant cytokine secretion capacity and strong antitumor capability [22,23,24]. Human γδT cells can be divided into three populations according to the variable TCR-δ chain expression, namely Vδ1, Vδ2, and Vδ3. Vδ2 cells, which constitute the majority of the peripheral blood γδT cells [25], are paired exclusively with the γ9 chain of the TCR; they inhibit proliferation, angiogenesis, lymphangiogenesis, and promote cancer cell apoptosis. γ9δ2T cells recognize unconventional antigens including stress molecules like MICA/B by the NKG2D receptor [26]. γδT cells expressing δ1 chain are mainly confined to the intraepithelial layer of the mucosal surface where they are involved in maintaining epithelial tissue integrity [27]. Vδ3 T cells constitute about 0.2% of peripheral blood cells in healthy humans; they are located mainly in the liver and the gut epithelium, thus are rarely studied in cancer [28]. γδT cells show structural heterogeneity, but, also, they possess some degree of plasticity in terms of their functional activity upon activation [29,30]. Upon activating reaction with a specific antigen, γδT effector cells can play an antitumor role by secreting cytokines, act through antibody dependent cellular cytotoxicity (ADCC), as well as other processes [31,32]. In contrast, γδ Treg cells show immunosuppressive properties promoting cancer growth, by impairing the function of various effector cells [33,34]. A particular subpopulation of γδ Treg17 cells are pro-inflammatory regulatory T cells defined by their production of interleukin 17 (IL-17). In a variety of cancer types, γδT17 cells promote the accumulation and expansion of immunosuppressive cells and promote the development of cancer [35]. Depending on the cytokine stimulation to which γδT cells are subjected they can switch from a protumor to an antitumor phenotype through the process called polarization [29,30]. Concerning the specific Vγ9 Vδ2 T cells subset, it has been reported that they can be polarized into γδT17 cells (producing only IL-17), γδ T1/17 cells (producing both IFN-γ and IL-17), γδT1 cells (producing both IFN-γ and TNF-α), and γδ T2 cells (producing IL-4) [36]. Their polarization towards each of these specific phenotypes requires distinct cytokine stimulations.

Immunotherapy approaches based on γδT cells involve either their adoptive transfer or stimulation/expansion of endogenous γδT cells in vivo.

The former consists of ex vivo expansion of γδT cells sourced from the PBMCs, using synthetic phosphoantigens or N-bis, followed by administration of the cultured γδT cells to the patient [37].

The latter relies on systemic administration of either phosphoantigens, nitrogen-containing bisphosphonates (N-bis), or zoledronate (ZA). Although the safety of immunotherapy with the γδT cells has been widely demonstrated in large clinical trials, the problem of the limited clinical efficacy still remains.

Studies report that this limitation could be related to activation-induced γδT cell energy, as well as decreased number of peripheral blood γδT cells after infusion of the stimulants [38]. Since effector function of the γδT cell can shift drastically from protumor to antitumor, the cytokine balance is absolutely critical in the tumor immune microenvironment. New strategies to exploit the ability of cytokines such as IL-21, IL-36γ, IL-12, IL-18, and IL-15 [38] to inhibit the protumor activity of γδT cells could significantly improve their anti-tumor efficacy [39,40].

### 2.2. Rationale for Engineering γδT Cells with CAR

With their in vitro, potent MHC-unrestricted lytic activity against a wide variety of tumor cells and their demonstrated clinical safety, γδT cells present as an attractive T cell subset to apply the CAR strategy.

**Figure 1:**
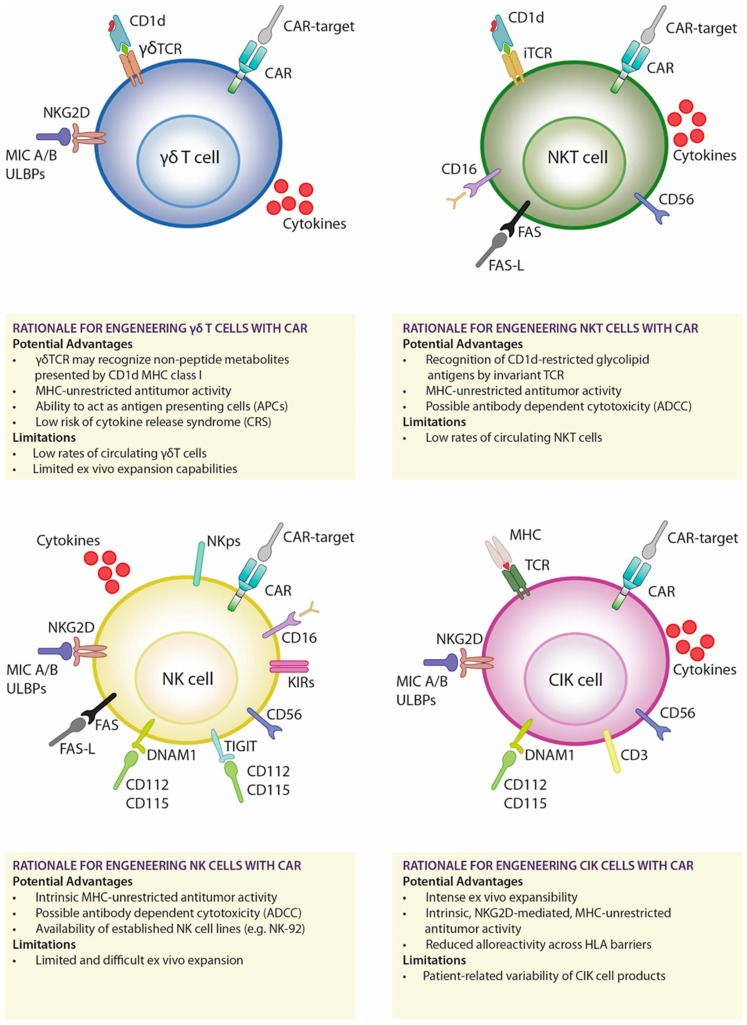
Main potential advantages and limitations of alternative cell types for CAR engineering. MHC, major histocompatibility complex; HLA, human leucocyte antigen; TCR, T cell Receptor; CAR, Chimeric Antigen Receptor; CD, Cluster of Differentiation; NKG2D, Natural Killer Group 2D receptor; MIC A/B, MHC class I chain-related gene A/B; ULBPs, UL16 binding proteins; KIR, killer immunoglobulin-like receptor; TIGIT, T cell Immunoglobulin and ITIM domain; DNAM-1, DNAX Accessory Molecule-1; NK, natural killer; CIK, Cytokine Induced Killer; APC, antigen-presenting cell; CRS, Cytokine Release Syndrome; ADCC, antibody dependent cellular cytotoxicity.

CAR-redirected γδT cells could combine both TCR-mediated cytotoxicity and MHC-unrestricted anti-tumor activity which is already an intrinsic property of the γδT cells. The antitumor potential of γδT cells could be synergized with standard chemotherapy or zoledronic acid (ZA) treatment, enhancing the potential of CAR-mediated attack. After chemotherapy and ZA treatments, tumor cells and the protective tumor microenvironment displayed altered metabolism and higher expression of isopentyl pyrophosphate [41], which increases susceptibility of γδ TCR-mediated recognition and killing [42]. This particular biological feature may be particularly relevant in the challenging setting of the solid tumors where the protective microenvironment is a limiting factor of CAR T cell therapy. A summary of the main features of γδT cells are reported in Figure 1.

### 2.3. Safety and Persistence

The use of CAR γδT cells could limit current CAR T-related toxicity. Firstly, low secretion of IL-2, IFNγ, and TNF, compared to αβT cells, considerably reduces the risk of cytokine release syndrome [43]. Secondly, the use of CAR γδT cells would lower the risk of αβTCR -mediated off-target toxicity [44]. Major limitations and causes of treatment failure with CAR T strategies are the loss of the target antigen by cancer cells and the reduced persistence of the CAR T lymphocytes [45]. In the case of the former, CAR γδT cells could still maintain antitumor activity by their endogenous TCRs and other receptors, such as NKG2D [46]. In addition, CAR γδT cells would also preserve their ability to act as APCs, capable of presenting soluble antigens to conventional CD4+ and CD8+ T cells. Target antigen repertoire would be effectively increased, recognized by TCRs of CD4+ and CD8+ T cells promoting an endogenous antitumor response even in the absence of the given CAR target antigen [47]. In case of the latter, the reduced persistence of CAR γδT cells could be counteracted by possible multiple reinfusions given their ex vivo expansibility, and also considering the future possibility of allogeneic donor sources based on their reduced alloreactivity across HLA-barriers. Furthermore, intriguing evidence supporting the existence of memory-like γδT cell subsets [48] provide new research opportunities to improve the applicability of γδ CAR-based approaches.

### 2.4. Preclinical Data and Ongoing Clinical Studies

Compared to a large number of preclinical studies on conventional CAR αβT cells, there are relatively few reports on CAR γδT cells. CAR γδT cells were first described in 2004, where Vγ9Vδ2 T cells were first successfully expanded, transduced with the chimeric receptor genes, and maintained in culture for a prolonged period of time. In this study authors demonstrated that γδT cells engineered with first-generation CAR-targeting GD2 (which is an antigen normally expressed by the neuroblastoma and Ewing sarcoma tumor cells) showed enhanced antigen-specific tumor reactivity. Antigen specific IFN-γ secretion and cytotoxicity against GD2+ neuroblastoma cell line LAN-1 was significantly higher compared to non-transduced zoledronate-expanded Vγ9Vδ2T cells [49]. γδT cells expressing the CD19ζ CAR co-cultured with CD19+ cell lines (Daudi, Raji, and Reh) produced similar results with substantial increases in target-dependent IFNγ production in mixed populations of CD19ζ+/− γδT cells [49]. Aforementioned preclinical studies suggest that CAR-expressing γδT cells might serve as potent and specific antitumor effector cells. Deniger and colleagues demonstrated that the propagation of CD19+CD64+CD86+CD137L+IL-15+ APCs enhanced the activity of anti-CD19 CAR γδT cells against preclinical leukemia models in vivo [50]. The ability of γδT cells to exert specific effector functions was not correlated with a particular Vδ or Vγ expression, as cells with different VδTCR frequencies produced the same cytokines and displayed similar cytolysis of CD19+ targets. It is yet to be elucidated whether CAR design for γδT cells requires optimization of γδTCR molecular structure and its co-stimulatory signals. Other preclinical studies reported that γδT cells expressed a series of co-stimulatory molecules such as CD28, CD27, and 4-1BB (CD137) [51,52]; all of them are involved in promoting survival, proliferation, and activation of human γδT cells [53].

Overall the accumulating evidence provides a solid rationale on exploiting CAR γδT cells as a possible strategy in cancer therapy.

#### Ongoing Clinical Studies

Currently there are not active clinical trials based on CAR γδT cells.

## 3. CAR NKT

### 3.1. Biological Features and Therapeutic Potential of NKT

Natural killer T cells are a population of immune cells that physiologically represent only a small percentage of circulating lymphocytes. These cells are essential not only for the defense against pathogens but also for the initiation and regulation of the adaptive and also autoimmune responses [54]. In contrast to typical T lymphocytes, NKT cells express an invariant αβ T cell receptor (TCR) that can recognize the antigen presented by the atypical MHC Class I molecule CD1D [55]. NKT cells have typical NK cell features, such as the Fc receptor CD16, CD56 expression, and granzyme production. These cells derive from the same lymphoid precursor as T cells and mature in the thymus [56].

We distinguish two types of NKT cells that differ in terms of TCR diversity and antigen specificities. Type 1 NKT cells have a semi-invariant TCR (iTCR) formed by an α-chain Vα24-Jα18 paired with a Vβ11 chain that recognizes both self and foreign glycolipid antigens presented by CD1d α-MHC class 1-like molecules such as galactosylceramide (α-GalCer), overexpressed by transformed cells [57].

Based on the surface expression, type I can be further subdivided into CD4+, CD8+, and double negative CD4−CD8−(DN) [58,59,60,61]. CD4+ NKT cells generally have a broader repertoire of responses when stimulated with the pro-inflammatory cytokines such as IL-12 or IL-2 [59,60,61]. CD4+ NKT cells secrete Th1 and Th2 cytokines, as well as perforin. In contrast, CD4− NKT cells can only secrete the Th1 cytokine and are responsive to PMA/ionomycin stimulation with perforin upregulation [61].

NKT type II, also known as diverse or variant NKT cells, are generally difficult to identify and have more diverse αβTCRs that do not recognize alpha-Galactosylceramide (α-GalCer) but rather a range of hydrophobic antigens, including sulfatide [62], lysophosphatidylcholine [63], and even small aromatic (non-lipid) molecules [64]. NKT cells demonstrate a dual role in cancer and can either promote or suppress antitumor immune responses. NKT type II cells are also associated with the regulation and inhibition of tumor immunity in murine tumor models and patients [63,65,66,67].

Conversely, NKT type I cells enhance antitumor immunity and seem to play an important role in the protective immune responses against tumors in contrast to NKT type II. NKT type I cells are able to kill CD1-expressing transformed cells by perforin [68,69], granzyme B, Fas ligand (FasL) [70], or TNF-α-mediated cytotoxic pathways [71].

NKT type I cells can also activate and recruit both innate and adaptive immune cells such as dendritic cells (DCs), NK, B, and T cells through a rapid secretion of cytokines [72]. They can influence the tumor microenvironment by killing CD1d-expressing tumor-associated macrophages (TAMs) and inhibiting myeloid-derived suppressor cells that promote tumor progression [73,74].

### 3.2. Rationale for Engineering NKT with CAR

Considering the effective antitumor immunity via direct tumor lysis, cytokine modulation of effector cells, regulation of immunosuppressive cells, and high ex vivo expansion, NKT cells pose a potent effector for CAR strategies and an attractive alternative to (or integration with) CAR T cells. Due to the low number of circulating NKT cells, the first step toward the generation of CAR NKT cells would be their harvesting by way of the peripheral blood of cancer patients. An intriguing experimental alternative could also consider an allogenic donor source in light of the NKT-reduced alloreactivity across HLA barriers.

After the isolation, NKT cells may be expanded by IL-2 and activated with α-GalCer-pulsed autologous irradiated PBMCs, then transduced to generate CAR NKT [75,76].

In the future, NKT engineering with CARs could result in an enhanced antitumor efficacy in solid tumor settings, exploiting the synergism of CAR activity with their capability to kill immunosuppressive CD1d-expressing tumor-associated macrophages (TAMs) and myeloid-derived suppressor cells [73]. A summary of the main features of iNKT cells are reported in Figure 1.

### 3.3. Safety and Persistence

With regard to the safety, data related to CAR NKT cells are limited.

Similar to γδT cells, NKT cells could be a safer option as an effector because they express an invariant TCR, which recognizes primarily glycolipid structures presented by CD1d which are not associated with the autoimmunity

The risk of severe autoreactive toxicities observed in an autologous setting of CAR-modified T cells has not been observed in initial studies with autologous NKT cells [77,78]

In a phase I clinical trial, the adoptive transfer of ex vivo-expanded autologous NKT cells in patients with advanced and recurrent non-small-cell lung cancer has not reported toxicity [79].

In addition, donor-derived NKT cells reported absence of alloreactivity, offering prospective rationale that immunotherapy based on allogeneic CAR NKT could present a favorable toxicity profile [77].

Another safety benefit could originate from their limited in vivo persistence that may be turned into positive safety implications. Given their ex vivo expandability, multiple low-dose infusions of CAR NKT are able to be envisioned while their withdrawal will allow for potential side effects to be limited.

Simon et al. showed that CAR-transfected NKT cells produced much lower quantities of IL-6 and other cytokines involved in cytokine release syndrome (e.g., TNF and IFNγ) than the CAR-transfected CD8+ T cells, suggesting a reduced potential risk for CRS [80].

Future early-phase I clinical trials are necessary in order to assess the real safety profile of CAR NKT cells.

CAR NKT do not present a long in vivo persistence but it could be increased by inducing a higher membrane expression of CD62L. In a preclinical study demonstrating the antitumor activity of CD19 CAR NKT cells in a mouse lymphoma model, the authors also identified CD62L as a marker for a prolonged in vivo persistence. To produce CD62L-enriched NKT cells they generated an artificial APC that induced stronger NKT expansion and CD62L expression. The infusion of these cells into immunodeficient mice showed that CD62L+ NKT cells may persist and proliferate in vivo much longer than their CD62L-negative counterpart [55]. This approach might be exploited and incorporated in future experimental studies to generate CAR NKT cells.

### 3.4. Preclinical Data and Ongoing Clinical Studies

Studies using CAR-modified NKT cells have been relatively scant. Heczey et al. demonstrated the feasibility of isolation, transduction, and expansion of CAR NKT cells and their capability to maintain the CAR expression up to three weeks [76].

In the same study they reported a strong and specific in vitro cytotoxicity of ex vivo-expanded human primary NKT, modified with CAR specific for the ganglioside GD2, against both GD2+ tumor cells as well as CD1d+ M2 macrophages in vitro. Moreover, they showed the importance of CAR co-stimulatory endodomains on the cytokine expression profiles of CAR NKT cells. In this model, GD2 CAR NKT cells effectively localized at the tumor site and presented strong antitumor activity without inducing GvHD [76].

CAR NKT cells efficiently impaired tumor growth and prolonged survival in GD2-expressing neuroblastoma and B cell lymphoma xenograft models [76].

Recently Simon et al. described a novel preclinical approach for transient RNA-based CAR transfer in NKT cells. They reported that specific anti-CSPG4 CAR NKT cells had lower pro-inflammatory cytokine secretion levels and an equal specific cytotoxicity compared to conventional CAR T lymphocytes [80].

#### Ongoing Clinical Studies

Currently, two phase I clinical trials are ongoing and the results are not yet available. The first study (NCT03294954) is exploring the efficacy and in vivo persistence of autologous GD2-CAR NKT cells (GINAKIT) in patients with neuroblastoma. The second study (NCT03774654) is exploring whether donor-derived NKT cells transduced with a vector codifying for the anti-CD19 chimeric receptor (containing CD28 as a costimulatory domain and IL-15 improving in vivo persistence) might help in patients with CD19+ lymphoma or leukemia (Table 1).

## 4. CAR NK

### 4.1. Biological Features and Therapeutic Potential of NK Cells

Natural killer (NK) cells are innate immune cells involved in the first line of defense that protects the body from pathogen invasion and malignant transformation. These cells constitute approximately 10% of lymphocytes in human peripheral blood (PB). NK cells are capable of killing tumor cells, as well as producing cytokines without previous stimulation [81].

Most NK cells are founded in the PB, liver, spleen, bone marrow, and a small portion are also present in the lymph nodes [82,83,84].

NK cells are regulated by signals deriving from large activating receptors such as NKp30, p46, p44, p80, NKG2D, DNAM-1 Natural Killer Group 2D receptor (NKG2D), DNAX Accessory Molecule-1 (DNAM-1), Ly49 (D-H), and KIR (2DL4, 2DS1, 2DS2,2DS3, 2DS4). A negative regulation is instead operated by inhibitory receptors such as NKG2A, LILR, KLRG1, Ly49 (A-C-I-P) and KIR (2DL1, 2DL 2/3, 2DL5, 3DL1 and 3DL2) [85,86,87]. Among the activating receptors, NKG2D and e DNAM-1 recognize, respectively, the stress ligands MIC-A, MIC-B, CD112, and CD155 that are generally overexpressed on the tumor cells [88].

When activated these cells rapidly release cytokines and chemokines, such as IFNγ, present cytotoxicity mediated by perforin, granzyme, FAS–FAS ligand interactions, and the TNF-related apoptosis-inducing ligand pathway [89].

According to levels of CD56 and CD16 expression levels we can distinguish CD56^dim^ CD16^bright^ and CD56^bright^ CD16^dim.^ NK cells, differing in the phenotype, function capabilities and tissue localization [90]. Although CD56^bright^ CD16^dim^ NK cells represent only 10% of PB NK cells they are highly enriched in the lymphoid organs. CD56^dim^ CD16^bright^ NK cells represent the large majority of PB NK cells and are the main subset recruited at inflamed tissue sites [90,91].

The characteristic features of NK cells create a potent immune effector against cancer.

Notwithstanding, NK-based immunotherapeutic strategies have been limited given their short lifespan in vivo (from a few days up to a few weeks) [92]. To this end, interesting reports support that upon a specific cytokine stimulation, NK cells could become memory-like with a longer lifespan in vivo; partially addressing the issue of NK limited persistence in vivo [93]. Phase I clinical trials reported significant clinical efficacy of NK cells against both hematological and solid tumors [94].

NK cell activity is a tightly regulated process, potentially stimulated or downregulated depending on the signaling molecules [95,96]. Multiple approaches may be considered to enhance NK antitumor activity. These may include: Induction of CD16-mediated antibody-dependent cytotoxicity (ADCC), IFN-γ production by the use of tumor antigen-specific mAb, increasing cytolytic activity by blocking inhibitory signals with anti-KIR mAb, and administration of NK stimulatory cytokines [95,96].

On the opposite end, several mechanisms of immune evasion in the tumor microenvironment have been reported; those have been shown to either repress NK cell recruitment to the tumor site or downregulate antitumor NK cell function. The key molecules are soluble factors such as transforming growth factor-β (TGF-β), indoleamine 2,3-dioxygenase (IDO), IL-4, and prostaglandin E2 (PGE2) [97,98,99]. These can interfere with NK cell activation or induce downregulation of activating NK receptors, which are involved in the NK cell-mediated recognition and antitumor activity [97,98,99]. Autologous NK cells may be functionally inhibited by the interaction between self-HLA class I molecules on tumor cells and inhibitory NK receptors [97,98,99]. Considering that HLA downregulation may be a relatively common tumor immune-escape mechanism, such a biological feature could actually provide an advantage for NK-based immunotherapy. Allogeneic NK cells showed stronger tumor killing than autologous NK cells [100,101,102]. Moreover, allogeneic NK cells can be obtained from many sources, such as bone marrow, human embryonic stem cells, induced pluripotent stem cells, PB, and umbilical cord blood. With the gradual progression of cell cloning technology, many NK cell lines have been established, including KHYG-1, NK-92, NKL, NKG, and YT cells. Among these cell lines, NK-92 is the most widely used and it is the only cell line that has been approved by the US Food and Drug Administration (FDA) for phase I and II clinical studies [103]. NK-92 cells lack the CD16 receptor and; therefore, cannot mediate ADCC [103].

### 4.2. Rationale for Engineering NK Cells with CAR

NK cells have been proposed to have some potential advantages as CAR drivers compared with T cells [104]. First of all, CAR NK cells may conjugate the killing activity based on CAR-specific mechanisms with their spontaneous tumor cytotoxic capability, mediated by receptors such as NKp46, NKp44, NKp30, NKG2D, and DNAM-1 (CD226) [105]. They are also capable of antibody-dependent cell-mediated cytotoxicity (ADCC) via FcγRIII (CD16). In the challenging solid tumor setting, where CAR T cell therapy reported poor efficacy, aforementioned features may result as valuable and attractive alternatives [12].

Secondly, NK cells can be used as established cell lines; the most used is NK-92, which originated from a 50-year-old man with malignant non-Hodgkin’s lymphoma that was already proven successful. This NK cell line is a homogeneous cell population that can be easily expanded under good manufacturing practice standards (GMP) for broader clinical applications [106], enabling the “off-the-shelf” production of CAR-modified NK-92 cells. Due to their tumoral origin, prior to infusion NK92 must be irradiated.

### 4.3. Safety and Persistence

The safety of NK cells have been proposed to be superior compared to safety of conventional CAR T cells [104].

The adoptive transfer of expanded, activated autologous NK cells reported a limited clinical efficacy related to the inhibition by self-HLA molecules, while NK cells from an allogeneic source may hold potential to be developed as a valid alternative approach. To minimize the occurrence of GvHD, allogeneic NK cells may be obtained from HLA-matched or haploidentical donors [107]. Several preclinical and clinical studies reported that haploidentical and cord blood (CB) NK cell infusions do not cause GvHD, both in patients with hematologic and solid malignancies [108,109,110].

Ruggeri et al. suggested that selection of KIR ligand–mismatched donors could help enhance the NK antitumor efficacy, reducing the risk of KIR-mediated NK inhibition [111].

On the other hand, the endogenous NK cell receptor repertoire plays a key role from the perspective of safety profile, protecting autologous healthy tissues by engaging inhibitory receptors on NK cells and, consequently, also in the hypothesis of adoptively transferred CAR NK cells.

CAR NK cells may be potentially a safer alternative to CAR T due to a reduced risk of inducing CRS. The cytokines produced by NK cells differ from those produced by T cells; activated NK cells usually produce high levels of IFN-γ, whereas the cytokine storm induced by CAR T cells is mainly mediated by pro-inflammatory cytokines, such as TNF-α, IL-1, and IL-6 [112]. As already discussed for CAR NKT cells, also in the case of CAR NK the limited persistence in vivo may present positive implications from a safety perspective.

CAR NK cells may have a limited lifespan in vivo [113], supporting the need for repetitive infusions. The need for repetitive infusions may be addressed by the recently-improved methodologies for NK ex vivo expansion, exploring allogeneic bank-derived donor NK or NK cell lines [114].

A promising approach is the possibility to engineer CAR NK cells to constitutively secrete IL-15, allowing the proliferation in the absence of exogenous cytokines to be sustained without impairing CAR functionality. The in vivo presence of IL-15 co-transduced cells could be detected for several days, supporting the beneficial effects of IL-15 for the persistence of CAR NK cells [115,116].

Initial clinical data support the notion that NK persistence in vivo may be enhanced by employing Hi-Cy/flu-conditioning regimens inducing transient lymphopenia and endogenous surges of IL-15 [108]. There are also intriguing evidence of NK subsets capable of sustaining immunological memory within infectious and noninfectious settings [117]. These features may provide new research opportunities to explore their possible exploitation to improve CAR NK-based strategies. A summary of the main features of NK cells are reported in Figure 1.

### 4.4. Preclinical Data and Ongoing Clinical Studies

Several preclinical studies reported the in vitro and in vivo efficacy of CAR NK therapy in different settings. A preclinical study demonstrated that NK cells expressing a CD19-specific CAR, with either the CD3ζ chain alone or linked to the 4-1BB domain, efficiently killed CD19+ targets and autologous leukemic lines in vitro [118]. In general, the second generation of CARs in NK cells is more active than the first-generation CARs. Chang Yh et al. showed that NK cells expressing the chimeric receptor with NKG2D specificity showed enhanced cytotoxicity against a wide spectrum of tumor subtypes, with the best responses observed in Acute Lymphoblastic Leukemia (ALL), osteosarcoma, prostate carcinoma, and rhabdomyosarcoma [119]. Altvater et al. discovered that 2B4 co-stimulation led to an overall increase in functionality of CD19 and GD2-specific CARs and restored killing of autologous leukemic and neuroblastoma cells [120].

Nonetheless, comparing a CAR construct carrying either 4-1BB and 2B4 co-stimulation, or both domains, did not show differences in antitumor activity against GD2+ allogeneic Ewing sarcoma cells in vitro [120].

Topfer K et al. reported that NK cells expressing the prostate stem cell antigen (PSCA)-specific CAR with a DAP-12 signaling motif, showed a higher antitumor activity in vitro and completely eradicated PSCA+ tumor cells in mice compared to NK cells engineered with CAR carrying only the CD3ζ chain [121].

Additionally, Ye Li et al. demonstrated that iPSC-derived NK-CAR were able to efficiently inhibit tumor growth and prolong survival in an ovarian cancer xenograft model. Indeed, this anti-tumor activity was greater than iPSC-derived CAR T. Furthermore, they observed a lower toxicity for CAR NK compared with the CAR T treatment.

Other preclinical studies explored the antitumor activity of the human NK-92 cell line engineered with CARs. Two among these reported that NK-92 cells redirected with a CD20- or CD19-specific chimeric antigen receptor were able to kill primary lymphoma and leukemic cells [122]. In another study, Liu et al. demonstrated that NK-92 cells expressing an erbB2-specific CAR could significantly prolong the survival of mice inoculated with erbB2+ breast cancer cells and induce the loss of the primary tumor and lung metastases [123].

In the field of solid tumors, a great obstacle is represented by the tumor microenvironment that may suppress CAR NK cell activity. One of main hurdles is represented by the tumor growth factor beta (TGF-β) interfering with granzyme and perforin secretion and downregulating NKG2D receptors [124].

In order to overcome CAR NK cells suppression, Wang et al. designed a CAR consisting of a TGF-β receptor extracellular domain fused with the intracellular signaling machinery of the NKG2D receptor. NK-92 cells engineered with TGF-β/NKG2D CAR released a higher amount of IFNγ, showed a lytic capacity, inhibited Treg differentiation, exhibited an enhanced chemoattraction to TGF-b-releasing tumor cells, and inhibited tumor growth in a xenograft model of liver cancer [125]. Additional reports explored the activity of CAR NK cells in tumor xenograft models, including CAR targets of hematological cancers (e.g., CD19, CD20, CD138, and CS-1) [122,126,127,128,129] and solid tumors (e.g., HER2, EpCam, GD2, GPA7, PSCA, EGFR, and EGFRvIII) [115,120,130,131,132,133,134,135,136].

#### Ongoing Clinical Studies

The majority of ongoing clinical trials are exploring the clinical antitumor activity of CAR NK cells against hematological malignancies. Only three studies with CAR NK are ongoing in the solid tumor setting. In both cases results have not yet been released.

As an overview, a phase I study (NCT03690310) is assessing the safety and relative efficacy of CAR cord blood natural killer (CB-NK) cells engineered with CAR.CD19-CD28-zeta-2A-iCasp9-IL-15 in patients with relapsed/refractory CD19+ B lymphoid malignancies. A phase I/II clinical trial (NCT03056339) aims to test efficacy and safety of CB-NK redirected with CAR.CD19-CD28-zeta-2A-iCasp9-IL-15 in patients with B-cell lymphoma treated with a high dose of chemotherapy and stem cell transplantation. Three early phase I clinical trials, in which recruiting has not yet started, will investigate the safety and efficacy of anti CD19 (NCT03824964), CD22, and CD19/CD22 (NCT03056339) CAR NK cells in patients with relapsed refractory B cell lymphoma.

In solid tumors, ongoing studies include a pilot study of NKG2D ligand-targeted CAR-NK cells (NCT03415100), an early phase I study (NCT03692637) of anti-mesothelin CAR NK cells in patients with epithelial ovarian cancer, and an early phase I study (NCT03692663) of anti-Prostate Specific Membrane Antigen (anti-PSMA) CAR NK cells in patients with castration-resistant prostate cancer.

Five ongoing clinical trials are exploring the safety and feasibility of adoptive immunotherapy with NK-92-expressing CAR. Three studies are evaluating the safety of CAR-NK92 (against CD19 (NCT02892695), CD33 (NCT02944162), and CD7 (NCT02742727)). Two studies (CAR against MUC-1 (NCT03383978) and HER-2 (NCT02839954)) are instead ongoing against solid tumors (Table 1).

## 5. CAR CIK

### 5.1. Biological Features and Therapeutic Potential

CIK cells are heterogeneous ex vivo expanded T lymphocytes generated from peripheral blood mononuclear cells (PBMC) [137,138]. They present a mixed T/NK phenotype and are endowed with MHC-unrestricted antitumor activity, reported against several tumor types including the relevant subset of chemoresistant cancer stem cells (CSC) [139,140,141,142,143]. An important feature of CIK cells is the extreme simplicity and effectiveness of their ex vivo expansion. The protocol for the ex vivo expansion of CIK cells includes the culture of the PBMCs for three to four weeks, with the timed addition of IFN-γ, antibody (Ab) anti-CD3, and IL-2 [137,138,144].

The average final CIK expansion levels are usually variable, ranging from dozen- to a hundred-fold. At the end of the expansion, CIK cells appear as a heterogeneous T-cell population where the CD3+CD56+ subset represents the cell type with the highest killing ability and antitumor efficacy [137,145,146].

Tumor-killing activity of CIK cells is supposed to be mainly mediated by the interaction of their membrane receptor NKG2D with stress-inducible molecules, such MHC class I chain-related gene A/B (MIC A/B) and UL16 binding proteins (ULBPs) on target cells [27,147,148,149].

Their MHC-unrestricted tumor killing and their intense ex vivo expansion make CIK cells a potential valid alternative to conventional cytotoxic T lymphocytes for immunotherapy.

The clinical activity and safety profile of CIK cells was demonstrated in several clinical trials in both hematological and solid settings [150].

### 5.2. Rationale for Engineering CIK Cells with CAR

As already discussed for NK, CIK cells could be a valid alternative to CAR conventional T cells, mostly in the field of solid tumors where conventional CAR T cells present a poor therapeutic efficacy and relevant safety warnings [151]. Key biological features of CIK cells may help address some of the main issues in this direction. First, their intense and cost-effective ex vivo expansion, as obtaining an elevated number of immune effectors is a key issue for the successful clinical translation of CAR-adoptive immunotherapy. Second, their NKG2D-mediated antitumor capacity would add a CAR-independent intrinsic therapeutic capability, potentially useful in the setting of solid tumors. Finally, the excellent safety profile, reported in various preclinical and clinical studies even when explored in allogeneic settings across HLA barriers [152].

A summary of the main features of CIK cells are reported in Figure 1.

### 5.3. Safety and Persistence

Preclinical and clinical data support the high safety profile of CIK-based strategies even when employed in allogeneic hematopoietic transplant settings [153].

The preclinical antitumor activity of CIK cells, including initial reports after CAR engineering, was reported to be exclusively directed against malignant targets with irrelevant toxicities observed against non-tumoral controls, like PBMCs or fibroblasts [154,155]. In vivo murine models, employing bioluminescence imaging (BLI) technology, confirmed the localization of CIK to tumor sites with minimal trafficking and localization in the context of healthy organs [141,144,156]. A recent preclinical study correlated the in vivo antitumor response with the persistence of anti-EGFR CAR CIK [144].

The CIK safety profile was confirmed in the initial clinical trials, including the challenging settings of allogeneic CIK infusions after hematopoietic cell transplants, with observed a low risk for GVHD events [157,158]. Preclinical in vitro studies demonstrated that CIK cells maintained their ability to actively proliferate when stimulated across major HLA barriers [146].

Another important feature, of potential clinical relevance, is CIK independence on exogenous administration of IL-2, which may be responsible for severe systemic toxicities.

Thanks to their intense and cost-effective ex vivo expansibility, relevant doses of the final cell product may be obtained and cryopreserved [142]. This aspect may be clinically important, considering the limited lifespan and persistence in vivo of CIK cells [156]. From a safety perspective, such limited in vivo persistence may provide a lower risk for prolonged side effects facilitating their “first in human” clinical exploration.

This is an important difference, as conventional CAR T lymphocytes are mainly employed as a single infusion based on their capacity of in vivo proliferation and long-time persistence.

### 5.4. Preclinical Data and Ongoing Clinical Studies

Preclinical studies exploring CAR CIK antitumor ability are reporting promising results both in hematological and in challenging solid tumor settings.

Marin et al. reported that CIK engineered with a specific CD19-CAR (including CD28 or 4-1BB costimulatory domains fused with CD3ζ signaling domain) presented higher cytotoxicity potential against B-ALL models than the CIK cells engineered with a CAR containing disulfide adapter protein 10 (DAP10) or CD3ζ domains alone [159,160].

The high efficiency of CAR CIK against CD19, carcinoembryonic antigen (CEA), CD123, and CD33 against B cell leukemia, colon carcinoma, and acute myeloid leukemia was reported, respectively. Moreover, anti-CD33 and anti-CD123 CAR also showed an increased production of pro-inflammatory cytokines, such as IFN-γ, TNF-α, and TNF-β, compared to unmanipulated CIK cells [161,162]

Leuci et al. reported preclinical evidence of CAR CIK activity, directed against the CD44v6 target, versus several types of soft tissue sarcomas [154].

Ren et al. showed that anti-EGFRCAR significantly enhanced in vitro and in vivo anti-tumor activity of CIK cells against EGFR+ human cancer, with increased in vitro production of cytokines IFN-γ and IL-2 compared with unmodified controls [163].

Interestingly, Micheal et al. demonstrated that ErbB2-CAR redirection of CIK cells improves their in vitro cytotoxicity against pediatric ErbB2-positive soft tissue sarcomas, compared with unmodified CIK cells. In addition, the same study showed anti-tumor cytotoxicity of ErbB2-CAR CIK against three-dimensional tumor spheroid models [164].

Finally, the in vitro anti-tumor efficacy of CAR CIK therapy was demonstrated also against ovarian cancer and nasopharyngeal stem cell-like cells [165].

#### Ongoing Clinical Studies

Despite the advantages presented above, ongoing clinical studies with CAR CIK cells are very few.

Their efficient preclinical antitumor activity and their very high safety provide basis for their dedicated exploration in clinical studies.

Currently, the ongoing clinical trials (NCT03389035) aim to determine the recommended phase two dose and the safety of CAR CIK-CD19 in adult and pediatric patients with relapsed or refractory B-cell precursor acute lymphoblastic leukemia (Table 1).

## 6. Conclusions

The initial impressive successes of CAR T strategies, against selected hematological malignancies, has prompted an intense enthusiasm and research efforts to improve the safety of this approach and extend its application to the challenging field of solid tumors. CAR engineering of alternative immune effectors, different from αβ T lymphocytes, is emerging as a promising integrative option to be explored in future clinical studies. A crucial and desirable biological feature of such alternative effectors is the intrinsic antitumor potential, proper of effectors of the innate immune response. This may represent an added value in tumor settings with heterogeneous CAR Target expression, limiting the risk of tumor clonal escape. The limited in vivo persistence and incapacity to sustain prolonged immunological memory may appear as the main downsides of such non-CAR T effectors; however, it also provides a positive perspective in reducing the risks of prolonged toxicities.

## Figures and Tables

**Table 1:** Main ongoing clinical trials involving alternative cell types as platforms for CAR engineering. CAR, Chimeric Antigen Receptor; PSMA, prostate specific membrane antigen; HER2, erb-b2 receptor tyrosine kinase 2; MUC, mucin; NK, natural killer; CIK, Cytokine Induced Killer.

Agent	CAR Target	Combination	NCT Identifier	Phase	Setting
CAR NKT cells	GD2	/	NCT03294954	1	Neuroblastoma
	CD19	/	NCT03774654	1	Refractory B-Cell Non-Hodgkin Lymphoma, Refractory B-Cell Small Lymphocytic Lymphoma, Relapsed Adult Acute Lymphoblastic Leukemia (ALL), Relapsed Chronic Lymphocytic Leukemia (CLL), Relapsed Non-Hodgkin Lymphoma
CAR NK cells	CD22	/	NCT03692767	1	Refractory B-Cell Lymphoma
	CD19	/	NCT03690310	1	Refractory B-Cell Lymphoma
	PSMA	/	NCT03692663	1	Castration-resistant Prostate Cancer
	NKG2D-ligands	/	NCT03415100	1	Metastatic Solid Tumors
	CD19/CD22	/	NCT03824964	1	Refractory B-Cell Lymphoma
	CD19	Chemotherapy	NCT03056339	1–2	B-Lymphoid Malignancies, Acute Lymphocytic Leukemia (ALL), Chronic Lymphocytic Leukemia (CLL), Non-Hodgkin Lymphoma
	Mesothelin	/	NCT03692637	1	Epithelial Ovarian Cancer
	CD19	High-Dose Chemotherapy, Stem Cell Transplant	NCT03579927	1–2	Mantle Cell Lymphoma, Recurrent Diffuse Large B-Cell Lymphoma, Recurrent Follicular Lymphoma, Refractory B-Cell Non-Hodgkin Lymphoma, Refractory Diffuse Large B-Cell Lymphoma, Refractory Follicular Lymphoma
CAR NK-92 cells	CD19	Stem Cell Transplant	NCT02892695	1–2	Acute Lymphocytic Leukemia, Chronic Lymphocytic Leukemia, Follicular Lymphoma, Mantle Cell Lymphoma, B-Cell Prolymphocytic Leukemia, Diffuse Large Cell Lymphoma
	CD33	/	NCT02944162	1–2	Acute Myelogenous Leukemia, Acute Myeloid Leukemia, Acute Myeloid Leukemia With Maturation, Acute Myeloid Leukemia Without Maturation, Acute Lymphocytic Leukemia (ALL)
	CD7	/	NCT02742727	1–2	Acute Myeloid Leukemia, Precursor T-Cell Lymphoblastic Leukemia-Lymphoma, T-Cell Prolymphocytic Leukemia, T-Cell Large Granular Lymphocytic Leukemia, Peripheral T-Cell Lymphoma, Angioimmunoblastic T-Cell Lymphoma, Extranodal NK/T-Cell Lymphoma (Nasal Type), Enteropathy-type Intestinal T-Cell Lymphoma, Hepatosplenic T-Cell Lymphoma
	HER2	/	NCT03383978	1	Glioblastoma
	MUC1	/	NCT02839954	1–2	Hepatocellular Carcinoma, Non-Small Cell Lung Cancer, Pancreatic Carcinoma, Triple-Negative Invasive Breast Carcinoma, Malignant Glioma of Brain, Colorectal Carcinoma, Gastric Carcinoma
CAR CIK cells	CD19	/	NCT03389035	1–2	Acute Lymphoblastic Leukemia in Relapse post Hematopoietic Stem Cell Transplantation (HSCT)
